# Nattokinase Supplementation and Cardiovascular Risk Factors: A Systematic Review and Meta-Analysis of Randomized Controlled Trials

**DOI:** 10.31083/j.rcm2408234

**Published:** 2023-08-15

**Authors:** Xinmin Li, Junzi Long, Qian Gao, Mengyang Pan, Jing Wang, Fangjie Yang, Yasu Zhang

**Affiliations:** ^1^School of Traditional Chinese Medicine, Henan University of Chinese Medicine, 450046 Zhengzhou, Henan, China; ^2^School of Rehabilitation Medicine, Henan University of Chinese Medicine, 450046 Zhengzhou, Henan, China

**Keywords:** nattokinase, cardiovascular disease, cardiovascular risk factor, meta-analysis

## Abstract

**Background:**

As a fibrinolytic enzyme from fermented soybean, nattokinase has been shown to be potentially beneficial for cardiovascular health, but current clinical evidences regarding the nattokinase supplementation on cardiovascular risk factors are various. This study aims to evaluate the cardiovascular efficacy of nattokinase.

**Methods:**

Four electronic databases were systematically searched to collect eligible randomized controlled trials. Data were extracted and summarized in a pre-designed form by two independent reviewers. Review Manager 5.4 software (Cochrane Library Software, Oxford, U.K.) was used for meta-analysis and bias risk assessment.

**Results:**

Six studies were eligible for quantitative analysis with 546 participants. The overall methodological quality of included studies was high. Relatively low total dosage of nattokinase had a negative effect on blood total cholesterol (MD [mean difference] = 5.27, 95% CI [confidence intervals]: 3.74 to 6.81, *p* < 0.00001), high-density lipoprotein cholesterol (MD = –2.76, 95% CI: –3.88 to –1.64, *p* < 0.00001), and low-density lipoprotein cholesterol (MD = 6.49, 95% CI: 0.83 to 12.15, *p* = 0.02). Nattokinase supplementation significantly reduced systolic blood pressure (MD = –3.45, 95% CI: –4.37 to –2.18, *p* < 0.00001) and diastolic blood pressure (MD = –2.32, 95% CI: –2.72 to –1.92, *p* < 0.00001), and led a slight increase in blood glucose (MD = 0.40, 95% CI: 0.20 to 0.60, *p* < 0.0001) as compared to placebo. Nattokinase group with relatively high total dosage also had a higher total cholesterol (MD = 3.18, 95% CI: 2.29 to 4.06, *p* < 0.00001) than control interventions, but no significant differences were found in levels of high-density lipoprotein cholesterol and low-density lipoprotein cholesterol. No significant correlation was found between nattokinase supplementation and triglyceride (*p* = 0.71). No notable adverse events were reported in all studies due to intake of nattokinase.

**Conclusions:**

This study further supports that nattokinase can be used as an effective adjunctive therapy for hypertension, but relatively low-dose supplementation of nattokinase may have no significant lipid-lowering effect. More work will need to be done to determine whether the positive efficacy of nattokinase on cardiovascular risk factors is dose-dependent.

**Systematic Review Registration:**

This work has been registered on PROSPERO (CRD42022315020).

## 1. Introduction

Cardiovascular diseases (CVDs) remain the most common causes of premature mortality and disability globally [1]. The major risk factors of CVD, such as coagulation abnormality, hypertension, dyslipidemia, and hyperglycemia have been well established [2]. Dietary modification is a fundamental strategy for the prevention of CVD, and adequate dietary choices may promote cardiovascular health [3]. Previous research has established that dietary intake of soybeans is negatively associated with the risks of CVD [4], which shows the promise of soy food as a dietary therapy for CVD.

Over past decades, traditional Japanese diets have attracted growing attention because of substantially low CVD morbidity and the highest life expectancy of Japanese population [5]. Natto is a famous traditional Japanese food made from fermented soybeans, which contains a variety of functional ingredients, including nattokinase. As a serine protease produced by Bacillus subtilis, nattokinase has potential anti-coagulatory, thrombolytic, anti-atherosclerotic, lipid-lowering, and anti-hypertensive effects [6, 7]. In addition to these favourable cardiovascular profiles, nattokinase can be orally administered with inexpensive cost, proven safety and preventative efficacy [8]. Hence, nattokinase consumption is growing in both healthy and CVD individuals around the world, especially in Asian countries.

Nattokinase has a stronger fibrinolytic activity than plasmin *in vivo*, and can even hydrolyze fibrin directly [9]. Oral administration of nattokinase not only promotes the release of tissue plasminogen activator from vascular endothelial cells, but inhibits the level of plasminogen activator inhibitors [10, 11]. Recently, experimenters also provide a new insight that nattokinase is able to prevent arteriosclerosis and thrombosis by exerting anti-inflammatory, anti-oxidative stress and anti-apoptotic effects [6, 12, 13, 14]. In addition, a number of *in vitro* and animal experiments have established that nattokinase suppresses hypertension via inhibiting angiotensin-converting enzyme and plasma angiotensin II level [8, 15, 16].

Although recent review studies suggest that nattokinase is a promising alternative in the prevention and treatment of CVD [17, 18, 19], cardiovascular benefits of nattokinase, such as its lipid-lowering effect, remain controversial [20, 21, 22, 23]. This study thus aims to assess the efficacy of nattokinase on cardiovascular risk factors and to provide evidence-based recommendations for clinical decision-making.

## 2. Methods

This systematic review and meta-analysis followed the Preferred Reporting Items for Systematic Reviews and Meta-Analyses (PRISMA) recommendations [24], and the protocol had been registered on PROSPERO (CRD42022315020).

### 2.1 Search Strategy

Electronic searches for pertinent studies were conducted in PubMed, Web of Science, Embase, and Cochrane Library from their inceptions to February 14, 2023, using the keyword “nattokinase” in the title or abstract. In order to adequately identify eligible studies, no restrictions were placed on terms of cardiovascular risk factors. Two authors separately screened retrieved records, and discrepancies were resolved by consulting with other authors. Moreover, references of included literature were checked to find more eligible studies.

### 2.2 Selection Criteria

(Ⅰ) Type of study: prospective parallel-group randomized controlled trials (RCTs) with at least a 1-month follow-up.

(Ⅱ) Subjects: adults with or without established cardiovascular risk factors.

(Ⅲ) Intervention and control measures: comparison between nattokinase supplementation and placebo intervention without limitations on oral dosage and frequency.

(Ⅳ) Outcome measurements: blood lipids, blood pressure, blood glucose, metabolic factors, hemorheological parameters, coagulation indexes, and adverse effects.

Studies in which the outcome measurements could not be synthetically evaluated were included for qualitative analysis but excluded from the meta-analysis. This study did not include trials that observed fermented soybeans (natto) on cardiovascular risk factors, because fermented soybean product contained a high amount of nutrients, some of which had cardiovascular benefits (not just nattokinase). Furthermore, we excluded cohort studies, case control studies, case report/series, and review studies.

### 2.3 Data Extraction and Quality Assessment

Collected data were summarized in a pre-designed form by two authors, and any difference was solved by consensus, which included basic information of research, population characteristics, medication administration details, and outcome measurements. Quality appraisal was undertaken by two reviewers based on the Cochrane Collaboration’s tool, and another author made a final decision regarding any disagreement.

### 2.4 Data Synthesis and Analysis

This meta-analysis was performed using the RevMan 5.4 version software (Cochrane Library Software, Oxford, U.K.). Quantitative analyses were carried out if more than one study reported the same outcome data that were available and consistent on clinical grounds. Between-study heterogeneity was tested by the Q-test (Chi-square) and quantified by the *$I^{2}$* statistic, where a significant Q-test (*p* < 0.10) and value for *$I^{2}$* > 50% represented high heterogeneity among studies, and random-effect models were used. Otherwise, the fixed-effect model was utilized [25]. For continuous variables, mean difference (MD) was utilized to signify the degree of deviation between variables, and standardized mean difference (SMD) was used when mean value varied widely. We conducted sub-group analysis to investigate the potential sources of heterogeneity, and provided a narrative overview for unexplained substantial heterogeneity. The sensitivity analysis would be used to assess the robustness of our findings via excluding trials that were considered to have a high risk of bias in one or more domains. Publication bias was detected using funnel plot if more than 10 eligible studies were included in the corresponding meta-analysis [26]. The *p* value of <0.05 was defined as statistically significant, and 95% confidence intervals (CI) were calculated for all included data.

## 3. Results

### 3.1 Search Results

**Fig. 1.**
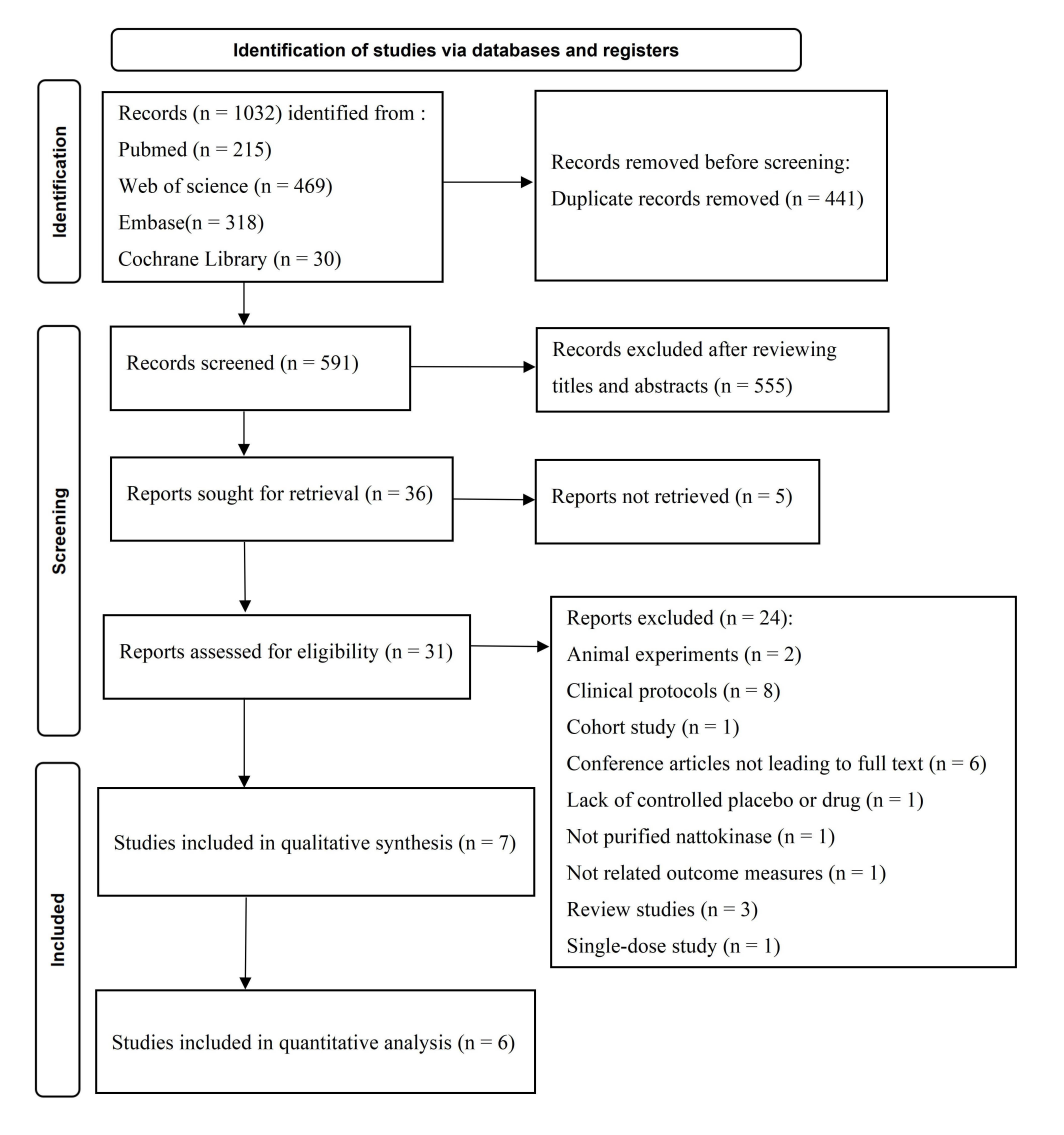
**The PRISMA flow diagram of study selection**.

The first search yielded 1032 studies, and thirty-six remained after excluding duplicates and irrelevant studies. Based on the selection criteria, 24 studies were removed, and 7 RCTs were finally included in the qualitative analysis [27, 28, 29, 30, 31, 32, 33]. Data from the 6 RCTs could be synthesized for the meta-analysis [27, 28, 29, 31, 32, 33]. The PRISMA flow chart presents the detailed retrieval process (Fig. 1).

### 3.2 Participant Characteristics

The main study characteristics are presented in Table 1 (Ref. [27, 28, 29, 30, 31, 32, 33]). A total of 311 participants received nattokinase and 296 received matching placebo. The mean number of participants per study was 86, ranging from 28 to 265. The average age of participants was significantly varied in each trial, and approximately 62% of the population was female. Five studies recruited subjects with cardiovascular disease risk factors, including hypertension [28, 29] and hyperlipidemia [31, 32, 33]. One trial recruited patients diagnosed with sub-acute ischemic stroke [30], and another trial included healthy subjects without any clinical evidence of cardiovascular risk factors [27]. Nearly 56% of the included participants were Americans and the others were Asians.

**Table 1.** **Characteristics of included trials evaluating the effect of nattokinase on risks of cardiovascular disease**.

Trial	Location	Study design	Participants	Age (year)	Nattokinase supplementation	Control	Outcomes for quantitative analysis
			N/C	Mean (SD)/[Range]			
Hodis *et al*. (2021) [27]	USA	RCT; prospective; single-center; double-blinded	132/133	65.3 [60.6–72.3]	2000 FU/day	Matching placebo	Total cholesterol, low-density lipoprotein cholesterol, high-density lipoprotein cholesterol, blood glucose
					36 months	36 months	
Jensen *et al*. (2016) [28]	USA	RCT; prospective; multi-center; double-blinded	39/35	53.4 [20.8–82.8]	2000 FU/day	Matching placebo	Blood pressure
					8 weeks	8 weeks	
Kim *et al*. (2008) [29]	South Korea	RCT; prospective; single-center; double-blinded	39/34	N: 47.6 ± 1.78	2000 FU/day	Matching placebo	Blood pressure
				C: 46.5 ± 1.65	8 weeks	8 weeks	
Pham *et al*. (2020) [30]	Vietnam	RCT; prospective; single-center; single-blinded	31/30	60.1 [30–70]	1200 FU/day	Matching placebo	Blood pressure
					60 days	60 days	
Wu *et al*. (2009) [31]	Taiwan	RCT; prospective; single-center; double-blinded	15/15	N: 54.8 ± 9.6	8000 FU/day	Matching placebo	Total cholesterol, triglyceride, low-density lipoprotein cholesterol, high-density lipoprotein cholesterol
				C: 51.6 ± 10.1	8 weeks	8 weeks	
Yang *et al*. (2009) [32]	Taiwan	RCT; prospective; multi-center; double-blinded	18/10	N: 51.6 ± 8.6	7000 FU/day	Matching placebo	Total cholesterol, triglyceride, low-density lipoprotein cholesterol, high-density lipoprotein cholesterol
				C: 56.3 ± 11.8	6 months	6 months	
Yoo *et al*. (2019) [33]	South Korea	RCT; prospective; single-center; double-blinded	37/39	N: 54.3 ± 1.25	6000 FU/day	Matching placebo	Total cholesterol, triglyceride, low-density lipoprotein cholesterol, high-density lipoprotein cholesterol, blood glucose
				C: 53.1 ± 1.40	8 weeks	8 weeks	

Abbreviations: N, nattokinase group; C, control group; FU, fibrinolytic unit; RCT, randomized controlled trial; SD, standard deviation.

### 3.3 Intervention and Outcome Measurements

In the present meta-analysis, nattokinase supplementation as an intervention to manage cardiovascular risk factors was compared with the matching placebo. The daily dosage of nattokinase was highly variable among the included trials, ranging from 1200 to 8000 FU (a fibrin unit used to quantify the ability of nattokinase to lyse fibrin *in vitro*) [34]. Both nattokinase and placebo were produced in the same capsule form. Five studies performed a follow-up evaluation at the eighth week [28, 29, 30, 31, 33]. The follow-up time of the other two studies was 6 months [32] and 3 years [27]. All included studies evaluated potential risks of CVDs, including blood coagulation and fibrinolysis factors, blood lipids, blood pressure, and blood glucose. Six of included trials observed adverse events encountered with nattokinase and control interventions [27, 28, 30, 31, 32, 33]. Four studies evaluated compliance by counting returned capsules [27, 28, 32, 33].

### 3.4 Risk of Bias Assessments

All the studies were randomized and provided information about randomization and allocation concealment. Double-blinded method was reported in six trials [27, 28, 29, 31, 32, 33] and one trial had a single-blinded design [30]. Two studies reported no patient drop-outs [29, 30], and five studies provided numbers and reasons for dropping out [27, 28, 31, 32, 33], so they all were considered to have low risk of attrition bias. All included trials reported the main results as planned, and four of them were judged to have a low risk of selective reporting bias [27, 29, 32, 33]. Table 2 (Ref. [27, 28, 29, 30, 31, 32, 33]) presents the risk of bias assessment results.

**Table 2.** **The summary of reviewers’ judgments about each risk of bias item for included trials**.

Trial	Random sequence generation	Allocation concealment	Blinding of participants and personnel	Blinding of outcome assessment	Incomplete outcome data	Selective reporting	Other bias
Hodis *et al*. (2021) [27]	L	L	L	L	L	L	L
Jensen *et al*. (2016) [28]	L	L	L	U	L	U	U
Kim *et al*. (2008) [29]	L	L	L	U	L	L	L
Pham *et al*. (2020) [30]	L	L	U	U	L	U	U
Wu *et al*. (2009) [31]	L	L	L	U	L	U	U
Yang *et al*. (2009) [32]	L	L	L	L	L	L	L
Yoo *et al*. (2019) [33]	L	L	L	L	L	L	L

Abbreviations: L, low risk of bias; U, unclear or unrevealed risk of bias.

### 3.5 Qualitative Results

*Blood coagulation and fibrinolytic parameters*. In a trial conducted among healthy subjects, nattokinase showed no detectable effects on any observed coagulation and fibrinolytic factor, such as prothrombin time, activated partial thromboplastin time, von Willebrand factor antigen and tissue plasminogen activator antigen, at time points of 1 week and 1, 3, and 6 months relative to placebo [27]. Jensen *et al*. [28] found that average level of von Willebrand factor was reduced by 15% in patients with hypertension after nattokinase supplementation, whereas consistent change was not found for subjects consuming placebo after 8 weeks (*p <* 0.09). In the study by Yoo *et al*. [33], nattokinase group had a greater increase in collagen–epinephrine closure time (*p* = 0.001) and activated partial thromboplastin time (*p* = 0.016) than those in placebo group.

*Degree of atherosclerosis*. Results of a 3-year intervention with nattokinase supplementation in healthy individuals showed that annualized rate of carotid artery intima-media thickness progression was 0.013 mm (95% CI, 0.010 to 0.015) per year in the nattokinase group, and 0.011 mm (95% CI, 0.009 to 0.013) per year in the placebo group (*p* = 0.31). In addition, the mean rate of carotid arterial stiffness was not significantly different between two groups [27].

*Blood pressure and associated regulators*. Pham *et al*. [30] found that nattokinase supplementation (60 days) led to statistically significant reductions (*p* < 0.05) in both systolic blood pressure (SBP) and diastolic blood pressure (DBP) compared to pre-treatment. In an 8-week trial, researchers found that patients’ DBP was significantly decreased after nattokinase intervention compared with placebo intervention (nattokinase: 84 ± 1.9 mmHg; placebo: 87 ± 1.7 mmHg; *p* < 0.01). Besides, participants with normal and high baseline plasma renin activity in both two groups had plasma renin activity decreased, and for subgroups with low baseline plasma renin activity, 66% of them in the nattokinase group had plasma renin activity improved to normal levels, but there were only 8% of subjects had plasma renin activity normalized in the placebo group [28]. During the 8-week intervention period, Kim *et al*. [29] found that the mean renin activity was increased in the control group and decreased in the nattokinase group without a statistically significant difference, but the corresponding net change was significant (*p* = 0.026).

### 3.6 Quantitative Data Synthesis and Analysis

#### 3.6.1 Effect of Nattokinase on Blood Lipids

Overall, four studies with a total of 399 participants included measures of total cholesterol, high-density lipoprotein cholesterol (HDL-C), and low-density lipoprotein cholesterol (LDL-C) [27, 31, 32, 33]. There was high heterogeneity for the above three meta-analyses (*$I^{2}$* = 52%, 95%, and 93%). We observed that subgroup-analysis based on the total dosages of nattokinase might explain the potential heterogeneity between these studies. Two trials had an 8-week nattokinase intervention [31, 33], and the other two observed nattokinase supplementation for 6 months and 3 years [27, 32], so the total therapeutic dose of these two subgroups were considered to be relatively low and high, respectively.

**Fig. 2.**
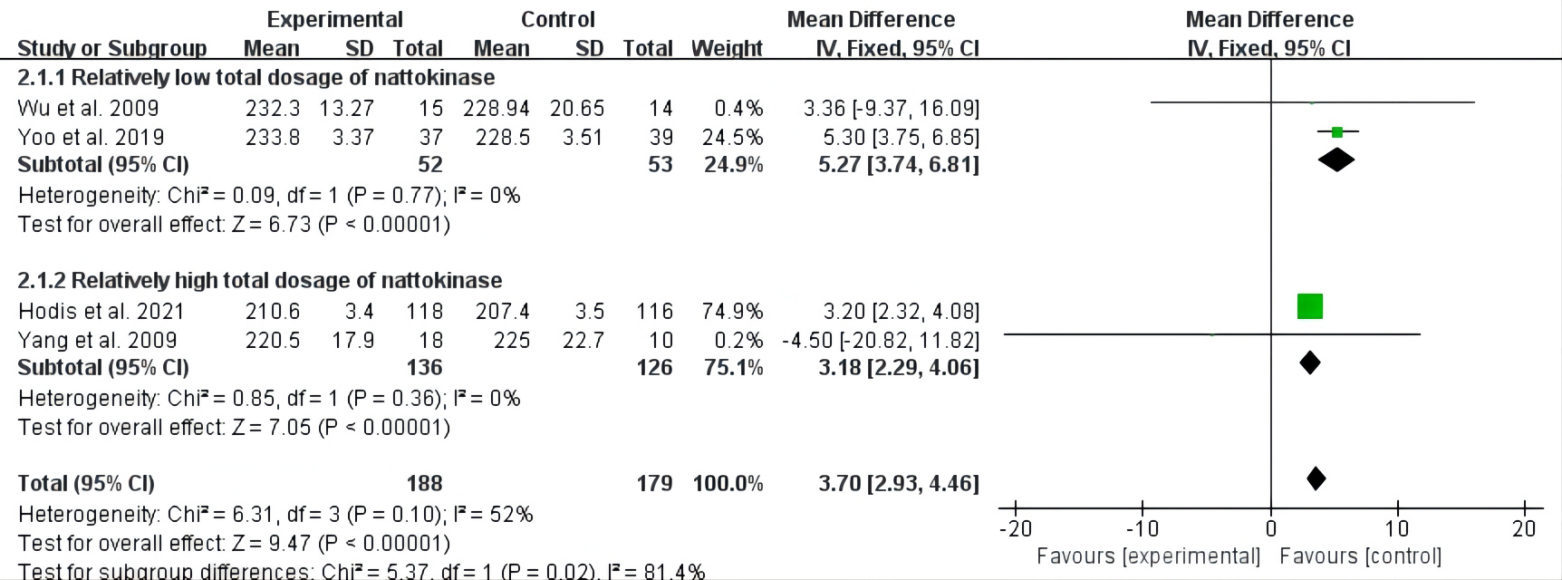
**Forest plot of RCTs investigating the effect of nattokinase on total cholesterol**. RCTs, randomized controlled trials; SD, standard deviation; CI, confidence intervals.

The subgroup of relatively low total nattokinase dosage showed that there was a positive association between nattokinase supplementation and total cholesterol (MD = 5.27, 95% CI: 3.74 to 6.81, *p* < 0.00001); relatively high total dosage of nattokinase supplementation also led to an increase in total cholesterol (MD = 3.18, 95% CI: 2.29 to 4.06, *p* < 0.00001) (Fig. 2).

**Fig. 3.**
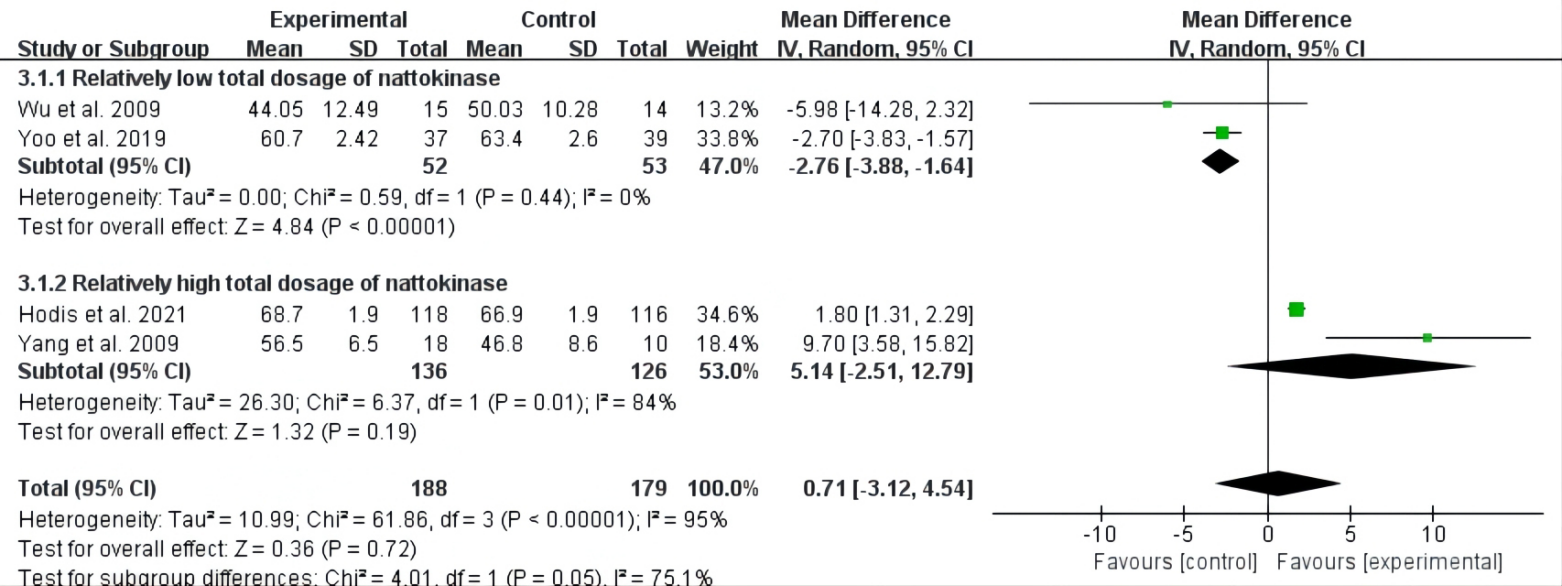
**Forest plot of RCTs investigating the effect of nattokinase high-density lipoprotein cholesterol**. RCTs, randomized controlled trials; SD, standard deviation; CI, confidence intervals.

**Fig. 4.**
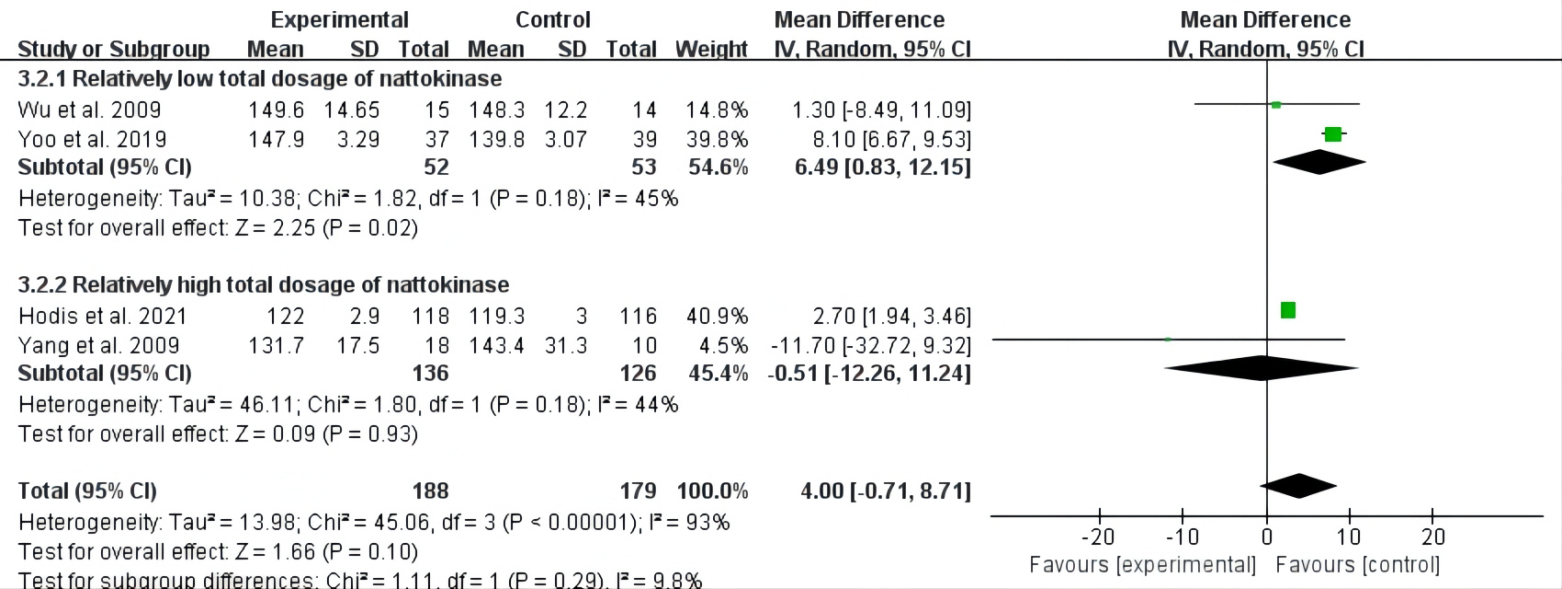
**Forest plot of RCTs investigating the effect of nattokinase on low-density lipoprotein cholesterol**. RCTs, randomized controlled trials; SD, standard deviation; CI, confidence intervals.

The aggregated results of these studies showed that nattokinase group with relatively low total dosage had more HDL-C levels reduced (MD = –2.76, 95% CI: –3.88 to –1.64, *p* < 0.00001) than control group. Nattokinase supplementation with relatively high total dosage showed a favorable but non-statistically significant effect in increasing HDL-C levels (MD = 5.14, 95% CI: –2.51 to 12.79, *p* = 0.19) compared with controls (Fig. 3). Furthermore, pooled analyses of the low total dosage subgroup showed that nattokinase induced higher LDL-C levels than control interventions (MD = 6.49, 95% CI: 0.83 to 12.15, *p* = 0.02), but no statistically significant effects were observed for the group with relatively high total nattokinase dosage (*p* = 0.93) (Fig. 4).

**Fig. 5.**
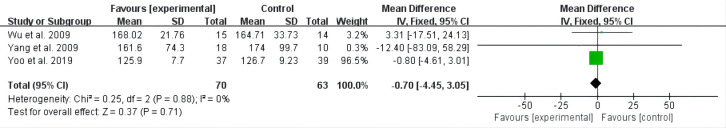
**Forest plot of RCTs investigating the effect of nattokinase on triglyceride**. RCTs, randomized controlled trials; SD, standard deviation; CI, confidence intervals.

As shown in Fig. 5, data from three studies with 134 participants were pooled to assess the effect of nattokinase on triglyceride [31, 32, 33]. No significant effect of intake of nattokinase was found in improving triglyceride levels (MD = –0.7, 95% CI: –4.45 to 3.025, *p* = 0.71).

#### 3.6.2 Effect of Nattokinase on Blood Pressure

**Fig. 6.**
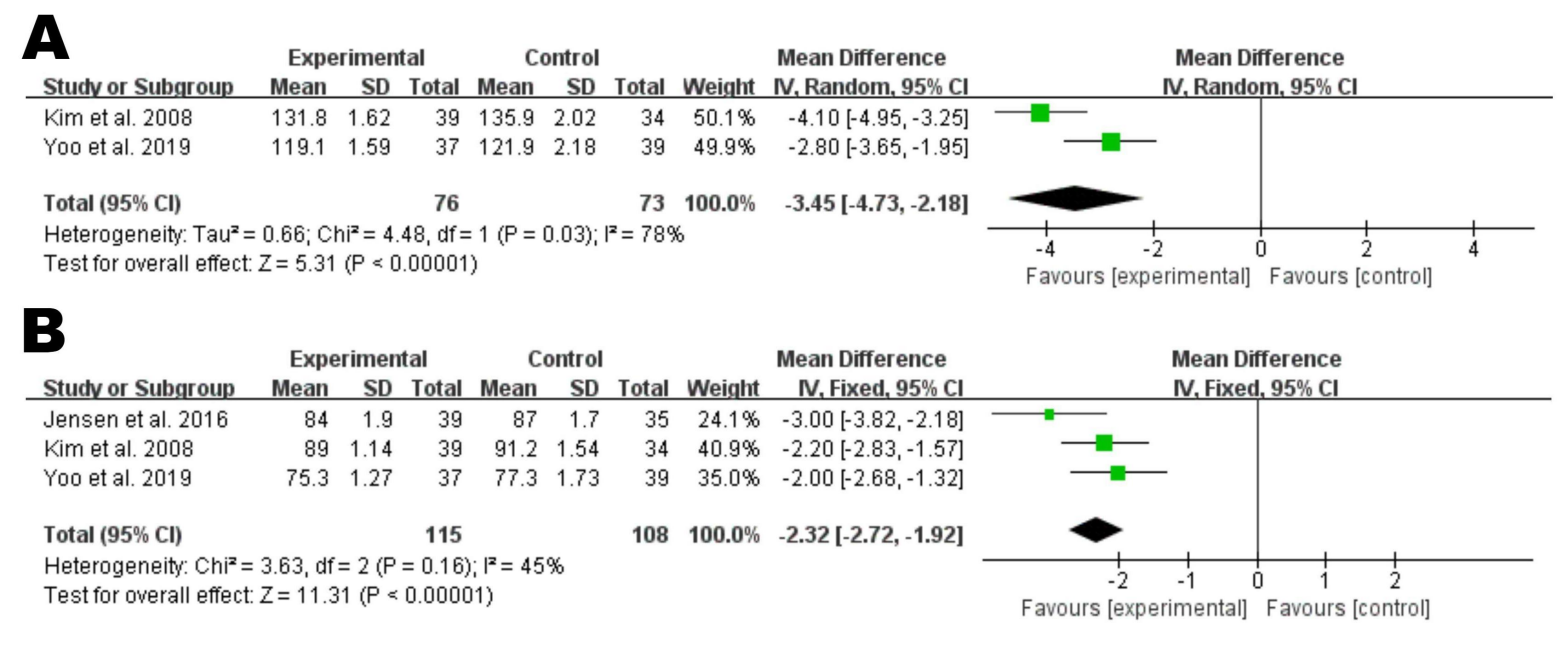
**Forest plot of RCTs investigating the effect of nattokinase on systolic blood pressure (A) and diastolic blood pressure (B)**. RCTs, randomized controlled trials; SD, standard deviation; CI, confidence intervals.

Based on the results of 3 RCTs that included 115 individuals in the nattokinase group and 108 individuals in the control group [28, 29, 33], nattokinase supplementation was found to be associated with a significant decrease in SBP (MD = –3.45, 95% CI: –4.37 to –2.18, *p* < 0.00001) and DBP (MD = –2.32, 95% CI: –2.72 to –1.92, *p* < 0.00001) as compared to placebo (Fig. 6).

#### 3.6.3 Effect of Nattokinase on Blood Glucose

**Fig. 7.**
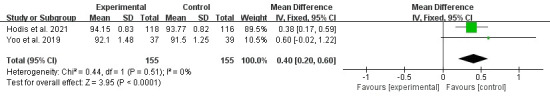
**Forest plot of RCTs investigating the effect of nattokinase on blood glucose**. RCTs, randomized controlled trials; SD, standard deviation; CI, confidence intervals.

The impact of nattokinase on blood glucose was evaluated in two studies, including a total of 341 participants (Fig. 7) [27, 33]. The results showed that nattokinase induced a slight increase of blood sugar with no heterogeneity across studies (*$I^{2}$* = 0%) in comparison with control interventions (MD = 0.40, 95% CI: 0.20 to 0.60, *p* < 0.0001).

### 3.7 Adverse Events and Compliance Rate

No notable adverse events were reported among all groups due to intake of nattokinase or placebo. The average compliance rate of participants exceeded 95% in three studies [27, 28, 32].

## 4. Discussion

To the best of our knowledge, this is the first systematic review to examine the association between nattokinase supplementation and cardiovascular risk factors. Overall, present study involving 607 participants found that nattokinase might have a beneficial influence on blood pressure, but no significant improvements were observed in blood lipids and blood glucose. Despite the high methodological quality of eligible studies in this meta-analysis, the total number of studies examining any cardiovascular risk factor was small, so these findings should be interpreted cautiously.

Since the fibrinolytic activity of nattokinase was discovered in the 1980s, a variety of animal-based studies have been made to support its strong thrombolytic activity [18, 35, 36, 37, 38]. Even a single dose of oral nattokinase was shown to enhance fibrinolysis and anticoagulation in humans [22, 39]. Moreover, nattokinase positively affected various blood rheological parameters in a dose-dependent manner, including platelet aggregation, red blood cell aggregation, whole blood viscosity and vascular tension, which could be considered as a good candidate in improving blood flow [40, 41, 42, 43]. Clinical data on fibrinolytic and antithrombotic effects of nattokinase were sparse in the present meta-analysis, which limits quantitative analysis of these results; however, it is worth mentioning that in recent years, nattokinase has been used on a much larger scale than previously—researchers have expanded their scopes to other cardiovascular benefits of nattokinase, such as lipid-lowering and hypotensive potentials.

In the meta-analysis of nattokinase on blood lipids, relatively low total dosage of nattokinase supplementation did not exert significant positive effects on levels of total cholesterol, LDL-C, and HDL-C, and even aggravated the dyslipidemia. A previous self-controlled clinical trial also reported no obvious effects of nattokinase (4000 FU/day, 8 weeks) on lipid parameters in both healthy volunteers and patients with cardiovascular risk factors [20]. Our findings were unexpected and inconsistent with results of several animal experiments [13, 44, 45]. This observation may be explained by the fact that all included trials used purified nattokinase products, whereas most previous animal studies used crude natto extracts that contained anti-cholesterol agents, such as soy isoflavones [32, 46].

However, a recent clinical study of 1062 patients with hyperlipidemia showed that nattokinase supplementation at a dosage of 10,800 FU per day for 1 year significantly decreased blood total cholesterol, triglyceride, LDL-C and increased HDL-C [47]. Ren *et al*. [48] also found that high-dose nattokinase administration over a relatively long period of time (26 weeks, 6500 FU) was effective in inhibiting the progression of atherosclerotic plaques and hyperlipidemia. Noticeably, in the meta-analysis of relatively high total dosage of nattokinase supplementation, the increase in HDL-C and the decrease in LDL-C were detected among included participants. So far, the lipid-lowering mechanism of nattokinase has not been understood, and one possible explanation is that nattokinase has proteolytic activity on some certain proteins involved in lipid metabolism [32]. In general, our observations reflected those of Chen *et al*. [17] who also proposed that long-term and high-dose nattokinase supplementation seemed to have positive impacts on blood lipids. For future researches, therefore, it is an important issue to determine if the lipid-lowering efficacy of nattokinase is dose-dependent.

Pooled results of this study suggested that nattokinase produced beneficial influences on CVDs by lowering SBP and DBP levels, which matched those observed in earlier animal studies [8, 15, 49]. It was already known that nattokinase had high gastrointestinal stability, and it might reduce blood pressure by cleaving plasma fibrinogen after absorption in the small intestine [8, 36]. More significantly, degradation products of nattokinase were demonstrated to exert different antihypertensive effects—inhibition of angiotensin I converting enzyme and plasma angiotensin II level [8, 16]. Results of the experiment by Ibe *et al*. [50] also indicated that nattokinase appeared to inhibit angiotensin converting enzyme related to increases in oral dosage. Renin is a key enzyme in renin-angiotensin system, which has long been considered as an attractive anti-hypertensive target [51]. Although researchers found a decrease in plasma renin activity in nattokinase group compared to controls, the results were not statistically significant [28, 29], and it was still not known whether nattokinase could prevent the elevation of plasma renin activity levels against arterial hypertension.

To date, data about the impact of nattokinase on blood sugar were limited. The present study found that nattokinase consumption had very little influence on blood glucose level (MD = 0.4). Combination of nattokinase, aronia, red ginseng, and shiitake mushroom was found to improve glucose metabolism and diminish insulin resistance [52]. Several randomized crossover studies also showed that breakfast accompanied with natto suppressed blood glucose elevation and improved insulin sensitivity in the early postprandial phase, but this may be attributed to γ-polyglutamic acid and polysaccharide components contained in the natto [53, 54, 55]. In a word, there is still no direct evidence to identify the antidiabetic effect of nattokinase.

In terms of safety, no major adverse events were reported for nattokinase supplementation in all included trials at different doses. A great deal of recent works corroborated that nattokinase was a safe agent for cardiovascular risk factors with low haemorrhagic risk and no toxicologic concerns. For instance, the standard safety margin (haemorrhagic adverse effect) of nattokinase was proven to be three times that of tissue plasminogen activators [56]. Daily nattokinase consumption of 10 mg/kg for 28 days was well tolerated in human volunteers [57], and even no adverse effects were observed when the daily dose of nattokinase was 480,000 FU/kg in mice, which was 1000-fold higher than the recommended daily dose in humans [58]. Nevertheless, several case studies have recently emerged that provide contradictory findings on allergic and bleeding risks of nattokinase [59, 60, 61]. As proposed by Gallelli *et al*. [23], therefore, although the positive effect of nattokinase on CVD outweighs possible described complications, patients must be always monitored for reference parameters, including clinical condition, coagulation profile, renal function, diet, and weight, and clinicians should make timely and reasonable dose adjustments to ensure the safety of nattokinase administered as monotherapy or in pharmacological combination.

## 5. Limitations

Several limitations of present study should be considered. Firstly, two major limitations were the small total sample size and varied therapeutic dosage of nattokinase for the included studies. Secondly, no restrictions were placed on the cardiovascular health status of included participants. Thirdly, quantitative analysis of the antithrombotic and anticoagulant effects of nattokinase was lacking. Fourthly, the heterogeneity in the corresponding meta-analyses was difficult to estimate because of limited included studies. Fifthly, our search strategies were limited to English papers, which might be linguistically biased. Last but not least, publication bias could not entirely be ignored, since less than 10 studies were pooled in the individual analysis.

## 6. Conclusions

Based on the available clinical data, the most obvious finding from this study was that short-term and low-dosage ingestion of nattokinase might have no significant lipid-lowering effects. The second major finding was that nattokinase could be considered as a promising adjunctive tool in the treatment of hypertension. Due to the existing limitations of this work, however, these findings could be considered to be preliminary, and a definitive conclusion on whether nattokinase supplementation was strongly associated with the improvement of any cardiovascular risk factor could not be drawn. Moreover, we inferred that the positive effect of nattokinase on cardiovascular risk factors might be enhanced with increasing oral doses, especially the hypolipidemic effect, and further clinical trials investigating the long-term and high-dose administration of nattokinase on cardiovascular risk factors were strongly recommended.

## Data Availability

The data used to support the results of this study are available from the corresponding author upon request.

## Supplementary Material

Supplementary material associated with this article can be found, in the online version, at https://doi.org/10.31083/j.rcm2408234.

## References

[1] Roth GA, Nguyen G, Forouzanfar MH, Mokdad AH, Naghavi M, Murray CJL (2015). Estimates of global and regional premature cardiovascular mortality in 2025. *Circulation*.

[2] Wilson PWF, Gagnon D (2017). Diabetes Mellitus and Control of Cardiovascular Disease Risk Factors: A Challenge to Improve Usual Care. *Circulation*.

[3] Shan Z, Li Y, Baden MY, Bhupathiraju SN, Wang DD, Sun Q (2020). Association Between Healthy Eating Patterns and Risk of Cardiovascular Disease. *JAMA Internal Medicine*.

[4] Yan Z, Zhang X, Li C, Jiao S, Dong W (2017). Association between consumption of soy and risk of cardiovascular disease: A meta-analysis of observational studies. *European Journal of Preventive Cardiology*.

[5] Niu K, Momma H, Kobayashi Y, Guan L, Chujo M, Otomo A (2016). The traditional Japanese dietary pattern and longitudinal changes in cardiovascular disease risk factors in apparently healthy Japanese adults. *European Journal of Nutrition*.

[6] Wu H, Wang Y, Zhang Y, Xu F, Chen J, Duan L (2020). Breaking the vicious loop between inflammation, oxidative stress and coagulation, a novel anti-thrombus insight of nattokinase by inhibiting LPS-induced inflammation and oxidative stress. *Redox Biology*.

[7] Dabbagh F, Negahdaripour M, Berenjian A, Behfar A, Mohammadi F, Zamani M (2014). Nattokinase: production and application. *Applied Microbiology and Biotechnology*.

[8] Fujita M, Ohnishi K, Takaoka S, Ogasawara K, Fukuyama R, Nakamuta H (2011). Antihypertensive effects of continuous oral administration of nattokinase and its fragments in spontaneously hypertensive rats. *Biological & Pharmaceutical Bulletin*.

[9] Fujita M, Hong K, Ito Y, Fujii R, Kariya K, Nishimuro S (1995). Thrombolytic effect of nattokinase on a chemically induced thrombosis model in rat. *Biological & Pharmaceutical Bulletin*.

[10] Yatagai C, Maruyama M, Kawahara T, Sumi H (2008). Nattokinase-promoted tissue plasminogen activator release from human cells. *Pathophysiology of Haemostasis and Thrombosis*.

[11] Urano T, Ihara H, Umemura K, Suzuki Y, Oike M, Akita S (2001). The profibrinolytic enzyme subtilisin NAT purified from Bacillus subtilis Cleaves and inactivates plasminogen activator inhibitor type 1. *The Journal of Biological Chemistry*.

[12] Chang CH, Chen KT, Lee TH, Wang CH, Kuo YW, Chiu YH (2010). Effects of natto extract on endothelial injury in a rat model. *Acta Medica Okayama*.

[13] Iwai K, Nakaya N, Kawasaki Y, Matsue H (2002). Antioxidative functions of natto, a kind of fermented soybeans: effect on LDL oxidation and lipid metabolism in cholesterol-fed rats. *Journal of Agricultural and Food Chemistry*.

[14] Wang Y, Wang H, Zhang Y, Xu F, Wang J, Zhang F (2022). Stepwise Strategy to Identify Thrombin as a Hydrolytic Substrate for Nattokinase. *Journal of Chemical Information and Modeling*.

[15] Lee BH, Lai YS, Wu SC (2015). Antioxidation, angiotensin converting enzyme inhibition activity, nattokinase, and antihypertension of Bacillus subtilis (natto)-fermented pigeon pea. *Journal of Food and Drug Analysis*.

[16] Murakami K, Yamanaka N, Ohnishi K, Fukayama M, Yoshino M (2012). Inhibition of angiotensin I converting enzyme by subtilisin NAT (nattokinase) in natto, a Japanese traditional fermented food. *Food & Function*.

[17] Chen H, McGowan EM, Ren N, Lal S, Nassif N, Shad-Kaneez F (2018). Nattokinase: A Promising Alternative in Prevention and Treatment of Cardiovascular Diseases. *Biomarker Insights*.

[18] Weng Y, Yao J, Sparks S, Wang KY (2017). Nattokinase: An Oral Antithrombotic Agent for the Prevention of Cardiovascular Disease. *International Journal of Molecular Sciences*.

[19] Li D, Hou L, Hu M, Gao Y, Tian Z, Fan B (2022). Recent Advances in Nattokinase-Enriched Fermented Soybean Foods: A Review. *Foods*.

[20] Hsia CH, Shen MC, Lin JS, Wen YK, Hwang KL, Cham TM (2009). Nattokinase decreases plasma levels of fibrinogen, factor VII, and factor VIII in human subjects. *Nutrition Research*.

[21] Hitosugi M, Hamada K, Misaka K (2015). Effects of Bacillus subtilis var. natto products on symptoms caused by blood flow disturbance in female patients with lifestyle diseases. *International Journal of General Medicine*.

[22] Kurosawa Y, Nirengi S, Homma T, Esaki K, Ohta M, Clark JF (2015). A single-dose of oral nattokinase potentiates thrombolysis and anti-coagulation profiles. *Scientific Reports*.

[23] Gallelli G, Di Mizio G, Palleria C, Siniscalchi A, Rubino P, Muraca L (2021). Data Recorded in Real Life Support the Safety of Nattokinase in Patients with Vascular Diseases. *Nutrients*.

[24] Page MJ, Moher D, Bossuyt PM, Boutron I, Hoffmann TC, Mulrow CD (2021). PRISMA 2020 explanation and elaboration: updated guidance and exemplars for reporting systematic reviews. *British Medical Journal*.

[25] Cumpston M, Li T, Page MJ, Chandler J, Welch VA, Higgins JP (2019). Updated guidance for trusted systematic reviews: a new edition of the Cochrane Handbook for Systematic Reviews of Interventions. *The Cochrane Database of Systematic Reviews*.

[26] Sterne JAC, Sutton AJ, Ioannidis JPA, Terrin N, Jones DR, Lau J (2011). Recommendations for examining and interpreting funnel plot asymmetry in meta-analyses of randomised controlled trials. *British Medical Journal*.

[27] Hodis HN, Mack WJ, Meiselman HJ, Kalra V, Liebman H, Hwang-Levine J (2021). Nattokinase atherothrombotic prevention study: A randomized controlled trial. *Clinical Hemorheology and Microcirculation*.

[28] Jensen GS, Lenninger M, Ero MP, Benson KF (2016). Consumption of nattokinase is associated with reduced blood pressure and von Willebrand factor, a cardiovascular risk marker: results from a randomized, double-blind, placebo-controlled, multicenter North American clinical trial. *Integrated Blood Pressure Control*.

[29] Kim JY, Gum SN, Paik JK, Lim HH, Kim KC, Ogasawara K (2008). Effects of nattokinase on blood pressure: a randomized, controlled trial. *Hypertension Research*.

[30] Pham PT, Han B, Hoang BX (2020). Nattospes as Effective and Safe Functional Supplements in Management of Stroke. *Journal of Medicinal Food*.

[31] Wu DJ, Lin CS, Lee MY (2009). Lipid-Lowering Effect of Nattokinase in Patients with Primary Hypercholesterolemia. *Acta Cardiologica Sinica*.

[32] Yang NC, Chou CW, Chen CY, Hwang KL, Yang YC (2009). Combined nattokinase with red yeast rice but not nattokinase alone has potent effects on blood lipids in human subjects with hyperlipidemia. *Asia Pacific Journal of Clinical Nutrition*.

[33] Yoo HJ, Kim M, Kim M, Lee A, Jin C, Lee SP (2019). The effects of nattokinase supplementation on collagen-epinephrine closure time, prothrombin time and activated partial thromboplastin time in nondiabetic and hypercholesterolemic subjects. *Food & Function*.

[34] Yang Y, Lan G, Tian X, He L, Li C, Zeng X (2021). Effect of Fermentation Parameters on Natto and Its Thrombolytic Property. *Foods*.

[35] Sumi H, Hamada H, Nakanishi K, Hiratani H (1990). Enhancement of the fibrinolytic activity in plasma by oral administration of nattokinase. *Acta Haematologica*.

[36] Fujita M, Hong K, Ito Y, Misawa S, Takeuchi N, Kariya K (1995). Transport of nattokinase across the rat intestinal tract. *Biological & Pharmaceutical Bulletin*.

[37] Kamiya S, Hagimori M, Ogasawara M, Arakawa M (2010). In vivo evaluation method of the effect of nattokinase on carrageenan-induced tail thrombosis in a rat model. *Acta Haematologica*.

[38] Suzuki Y, Kondo K, Matsumoto Y, Zhao BQ, Otsuguro K, Maeda T (2003). Dietary supplementation of fermented soybean, natto, suppresses intimal thickening and modulates the lysis of mural thrombi after endothelial injury in rat femoral artery. *Life Sciences*.

[39] Ero MP, Ng CM, Mihailovski T, Harvey NR, Lewis BH (2013). A pilot study on the serum pharmacokinetics of nattokinase in humans following a single, oral, daily dose. *Alternative Therapies in Health and Medicine*.

[40] Pais E, Alexy T, Holsworth RE, Meiselman HJ (2006). Effects of nattokinase, a pro-fibrinolytic enzyme, on red blood cell aggregation and whole blood viscosity. *Clinical Hemorheology and Microcirculation*.

[41] Jang JY, Kim TS, Cai J, Kim J, Kim Y, Shin K (2013). Nattokinase improves blood flow by inhibiting platelet aggregation and thrombus formation. *Laboratory Animal Research*.

[42] Ji H, Yu L, Liu K, Yu Z, Zhang Q, Zou F (2014). Mechanisms of Nattokinase in protection of cerebral ischemia. *European Journal of Pharmacology*.

[43] Watanabe N, Takaoka S, Uehara S, Ohta M (2018). Effect of nattokinase on the blood flow improvement in healthy subjects-a randomized, placebo-controlled, double-blind, cross-over study. *Japanese Pharmacology and Therapeutics*.

[44] Park KJ, Kang JI, Kim TS, Yeo IH (2012). The antithrombotic and fibrinolytic effect of natto in hypercholesterolemia rats. *Preventive Nutrition and Food Science*.

[45] Min Y (2014). Effects of nattokinase extraction on anti-thrombosis hypolipidemic and antioxidative function. *Master’s Thesis*.

[46] Huang C, Pang D, Luo Q, Chen X, Gao Q, Shi L (2016). Soy Isoflavones Regulate Lipid Metabolism through an AKT/mTORC1 Pathway in Diet-Induced Obesity (DIO) Male Rats. *Molecules*.

[47] Chen H, Chen J, Zhang F, Li Y, Wang R, Zheng Q (2022). Effective management of atherosclerosis progress and hyperlipidemia with nattokinase: A clinical study with 1,062 participants. *Frontiers in Cardiovascular Medicine*.

[48] Ren NN, Chen HJ, Li Y, Mcgowan GW, Lin YG (2017). A clinical study on the effect of nattokinase on carotid artery atherosclerosis and hyperlipidaemia. *Zhonghua Yi Xue Za Zhi*.

[49] Suwanmanon K, Hsieh PC (2014). Effect of γ-aminobutyric acid and nattokinase-enriched fermented beans on the blood pressure of spontaneously hypertensive and normotensive Wistar-Kyoto rats. *Journal of Food and Drug Analysis*.

[50] Ibe S, Yoshida K, Kumada K, Tsurushiin S, Furusho T, Otobe K (2009). Antihypertensive effects of natto, a traditional Japanese fermented food, in spontaneously hypertensive rats. *Food Science and Technology Research*.

[51] Bavishi C, Bangalore S, Messerli FH (2016). Renin Angiotensin Aldosterone System Inhibitors in Hypertension: Is There Evidence for Benefit Independent of Blood Pressure Reduction. *Progress in Cardiovascular Diseases*.

[52] Yang HJ, Kim MJ, Kwon DY, Kim DS, Zhang T, Ha C (2018). Combination of Aronia, Red Ginseng, Shiitake Mushroom and Nattokinase Potentiated Insulin Secretion and Reduced Insulin Resistance with Improving Gut Microbiome Dysbiosis in Insulin Deficient Type 2 Diabetic Rats. *Nutrients*.

[53] Taniguchi-Fukatsu A, Yamanaka-Okumura H, Naniwa-Kuroki Y, Nishida Y, Yamamoto H, Taketani Y (2012). Natto and viscous vegetables in a Japanese-style breakfast improved insulin sensitivity, lipid metabolism and oxidative stress in overweight subjects with impaired glucose tolerance. *The British Journal of Nutrition*.

[54] Araki R, Yamada T, Maruo K, Araki A, Miyakawa R, Suzuki H (2020). Gamma-Polyglutamic Acid-Rich Natto Suppresses Postprandial Blood Glucose Response in the Early Phase after Meals: A Randomized Crossover Study. *Nutrients*.

[55] Matsumoto Y, Takahashi M, Sekimizu K (2020). Polysaccharides of a fermented food, natto, suppress sucrose-induced hyperglycemia in an in vivo evaluation system and inhibit glucose uptake by human intestinal cells. *Drug Discoveries & Therapeutics*.

[56] Guo H, Ban YH, Cha Y, An ES, Choi J, Seo DW (2019). Comparative anti-thrombotic activity and haemorrhagic adverse effect of nattokinase and tissue-type plasminogen activator. *Food Science and Biotechnology*.

[57] Lampe BJ, English JC (2016). Toxicological assessment of nattokinase derived from Bacillus subtilis var. natto. *Food and Chemical Toxicology*.

[58] Wu H, Wang H, Xu F, Chen J, Duan L, Zhang F (2019). Acute toxicity and genotoxicity evaluations of Nattokinase, a promising agent for cardiovascular diseases prevention. *Regulatory Toxicology and Pharmacology*.

[59] Ramachandran L, Aqeel A, Jafri A, Sidhu Y, Mohamed Djirdeh T (2021). Nattokinase-Associated Hemoperitoneum in an Elderly Woman. *Cureus*.

[60] Awatani-Yoshidome K, Hashimoto T, Satoh T (2022). Anaphylaxis from nattokinase in a patient with fermented soybean (natto) allergy. *Allergology International*.

[61] Chang YY, Liu JS, Lai SL, Wu HS, Lan MY (2008). Cerebellar hemorrhage provoked by combined use of nattokinase and aspirin in a patient with cerebral microbleeds. *Internal Medicine*.

